# Clinical Effects of the Probing Method with Depth Gauge for Determining the Screw Depth of Locking Proximal Humeral Plate

**DOI:** 10.1155/2016/5898161

**Published:** 2016-11-15

**Authors:** Lin Jin, Jialiang Guo, Junfei Guo, Yingchao Yin, Zhiyong Hou, Yingze Zhang

**Affiliations:** Department of Orthopaedic Surgery, Third Hospital of Hebei Medical University, Shijiazhuang, Hebei 050051, China

## Abstract

*Background*. The use of locking plates has gained popularity to treat proximal humeral fractures. However, the complication rates remain high. Biomechanical study suggested that subchondral screw-tip abutment significantly increased the stability of plant. We present a simple method to obtain the proper screw length through the depth gauge in elderly patients and compared the clinical effects with traditional measuring method.* Methods*. 40 patients were separated into two groups according to the two surgical methods: the probing method with depth gauge and the traditional measuring method. The intraoperative indexes and postoperative complications were recorded. The Constant and Murley score was used for the functional assessment in the 12th month.* Results*. Operative time and intraoperative blood loss indicated no statistical differences. X-ray exposure time and the patients with screw path penetrating the articular cartilage significantly differed. Postoperative complications and Constant and Murley score showed no statistical differences.* Conclusions*. Probing method with depth gauge is an appropriate alternative to determine the screw length, which can make the screw-tip adjoin the subchondral bone and keep the articular surface of humeral head intact and at the same time effectively avoid frequent X-ray fluoroscopy and adjusting the screws.

## 1. Introduction

Proximal humeral fractures represent approximately 5% of all fractures and 10% of these patients are older than 65 years [1–4]. As the population ages there is an increase in the number of people in poor general condition with an increased risk of falls on fragile bones [5]. Although most patients can be treated by nonsurgical management [6], many patients require surgical treatment. With the development of fixation techniques, especially the locking plates, open reduction and internal fixation has gained popularity to treat proximal humeral fractures [5, 7, 8].

Patients treated with locking plate can be mobilized earlier and get better functional recovery. However, the complication rates remain high [9–12], such like failure of reduction, avascular necrosis, varus collapse, subacromial impingement, and screw cutout of the articular surface [10, 11, 13]. Due to the characteristics of osteoporosis, stable fixation with plate and screw of such fractures remains a challenge, and more and more attention has been attracted. Biomechanical study suggested that subchondral screw-tip abutment significantly increased the stability of plant [14]. Therefore, obtaining an ideal screw length is important. Obviously, short screw cannot afford enough holding force [14]. The long screw penetration of the humeral head can occur as an original complication or secondary to fracture settling caused by medial instability [15]. However, the incidence of original penetration into the glenohumeral joint has been reported up to 10%–15% [5, 13], which will cause the destruction and chondrolysis of the articular cartilage [16–18].

In the traditional surgical method, the depth of drilling mostly depended on the tactile feedback from the drill bit against the subchondral bone. Given the characteristics of osteoporotic patients, a surgical method that was called “sounding” was reported in the literature [19]. They firstly used a drill bit to perforate the lateral cortex of the desired locking screw slot, then the blunt tip of a Kirschner wire was used to be inserted by hand until feeling a firm end point which indicated the blunt tip against the subchondral bone, and thus a more accurate and safer depth was obtained. In our study, a depth gauge was used instead of Kirschner wire and we called it “probing method.”

A clinical trial was performed to compare the clinical effects of management of proximal humeral fractures with either our probing method with depth gauge or traditional surgical method in geriatric patients. We hypothesized that an ideal screw length and good bone purchase will be obtained through probing method.

## 2. Patients and Methods

The study was approved by the Ethics Committee of the Third Hospital of Hebei Medical University and all aspects of the study comply with the Declaration of Helsinki. Between August 2012 and February 2013, we treated 40 patients with proximal humeral fractures based on the following criteria: (1) age > 55; (2) acute fracture, and the patients received the treatment of surgery within 2 weeks after an injury; (3) osteoporosis. We excluded patients based on the following conditions: (1) inability to cooperate with the informed consent, such like mental disorders; (2) preexisting disabling disorder; (3) open or pathological fractures; (4) existing neurovascular impairment; (5) rehabilitation unable to be performed as required; and (6) contraindication to surgery.

The mean age was 64.95 (57–72), including 15 male patients and 25 female patients. 28 fractures were caused by falls, and another 12 fractures were caused by traffic injuries. Based on the Neer classification system [20], all the fractures contained 3 two-part fractures, 32 three-part fractures, and 5 four-part fractures.

Patients were randomly divided into two groups according to the following management strategies: traditional measuring method (20 patients) and probing method with depth gauge (20 patients). A standard surgical procedure was performed by two experienced orthopaedic trauma surgeons from our institution. The patient was placed supine on a radiolucent operating room table. A deltopectoral approach was used to expose the fracture. Once the fracture was reduced, the locking plate was positioned and provisionally stabilized. Depending on the fracture pattern, screw insertion may begin either proximally or distally. During the proximal locking screws insertion, a drill bit was utilized to perforate the lateral cortex of the desired locking screw slot firstly.

**Figure 1:**
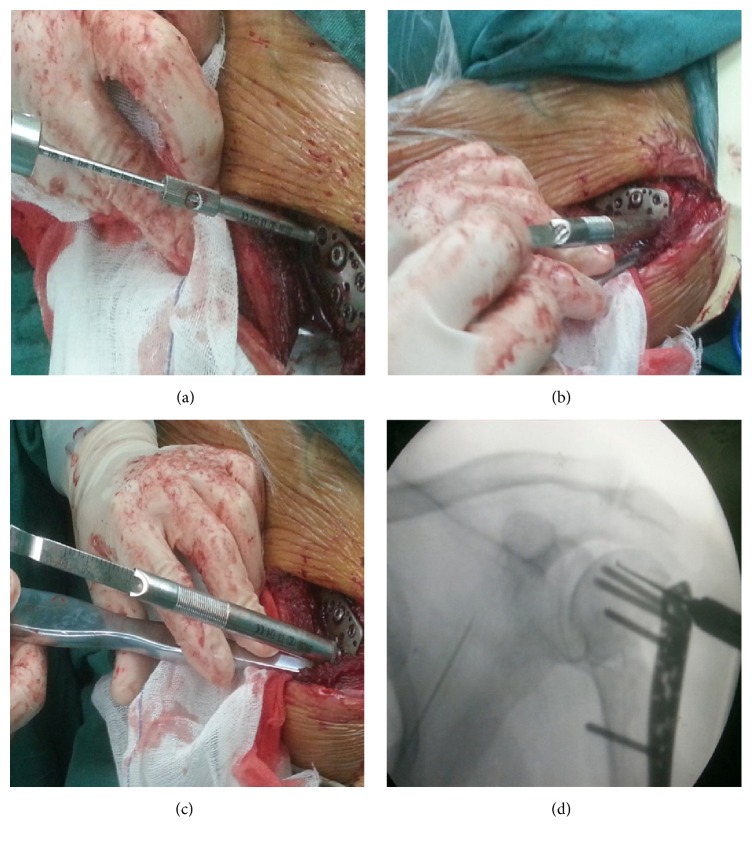
Probing method with depth gauge: (a) after perforating the lateral cortex, predrilling additional length of the cancellous bone with the locking drill sleeve to form a screw track with right direction; (b) removing the sleeve, the depth gauge was used to be advanced by hand; (c) a firm end point was felt, which indicated the tip reached the area close to subchondral bone; (d) verifying the position of the depth gauge with fluoroscopy.

In the traditional measuring method group, the drill bit advanced after perforating the lateral cortex and the depth of drilling was determined by the tactile feedback from the drill bit against the subchondral bone. In the probing method group, after perforating the lateral cortex, we predrilled additional length of the cancellous bone with the locking drill sleeve to form a screw track with right direction and then removed the sleeve; the depth gauge was used to be advanced by hand until a firm end point was felt, which indicated that the tip reached the area close to subchondral bone, and then we measured the depth directly. The position of the depth gauge tip can be verified with fluoroscopy (Figure 1). The locking plate was fixed by 4–6 proximal locking screws and 2-3 distal conventional screws. The lesser tubercle fracture or rotator cuff injury can be repaired by absorbable sutures. Rotator cuff sutures can be tied to the locking plate to enhance the stability.

The same postoperative rehabilitation protocol was performed for all the patients. Firstly, the arm was placed in a sling. Active mobilization of elbow, wrist, and hand may begin on the first day after surgery. Pendulum exercises could be carried out early. Once the patient was comfortable, gentle passive motion of the shoulder was started. Active motion of shoulder should start at 4 to 6 weeks, and strengthening exercises were carried out from 12 weeks on.

Operative time, blood loss, and X-ray exposure time (1 X-ray fluoroscopy was recorded as 1 second) were recorded. CT examination was routinely used to assess the screw length and detect whether there is a path through the articular surface. The screw path penetrating the articular cartilage was recorded. During 12-month follow-up, complications were recorded and the Constant and Murley score (CMs) [21] was used for the assessment of all patients in the 12th month by experienced orthopaedic surgeon. The CMs was graded as excellent (86–100), good (71–85), moderate (56–70), or poor (0–55) [21], and the fineness rate (the percentage of the excellent and good grades) was compared between the two groups.

The statistical analysis was performed using the SPSS 21.0 statistical software. 2-independent-sample *t*-test, Chi-square test, or Fisher exact test were applied to all outcome analyses, where appropriate. Statistical significance was defined as *p* < 0.05.

## 3. Results

**Figure 2:**
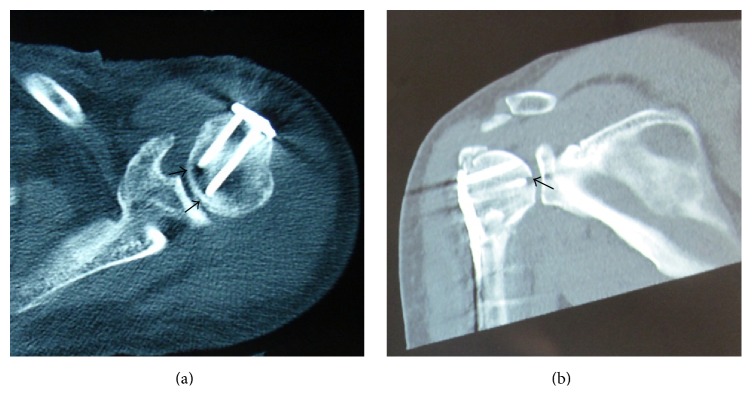
(a) Patient in the probing group without the articular cartilage disruption; (b) patient in the traditional group with screw path penetrating the articular cartilage, which was caused by accidental penetration during operation.

**Figure 3:**
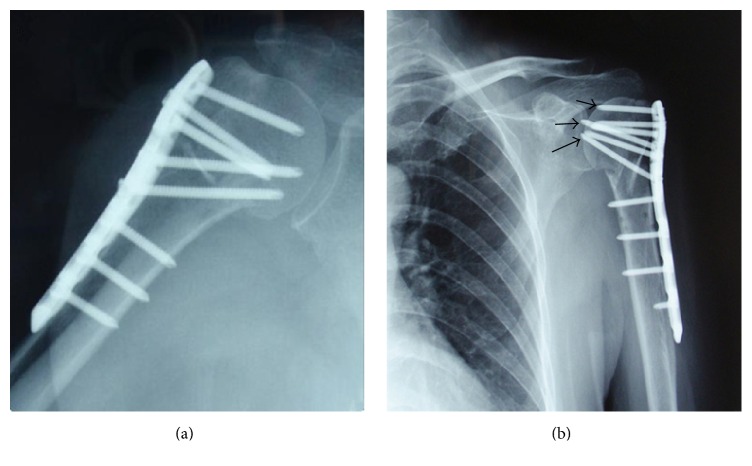
During the follow-up period, (a) patient from the probing group had a good screw length; (b) screw penetration complication occurred in the traditional measuring method group (black arrows).

Operative time (110.55 ± 9.36 min versus 107.05 ± 8.22 min, *p* = 0.217) and intraoperative blood loss (156.50 ± 33.13 mL versus 146.50 ± 29.78 mL, *p* = 0.322) indicated no statistical differences between two groups. X-ray exposure time (13.15 ± 2.13 s versus 11.50 ± 1.73 s, *p* = 0.011) and the patients with screw path penetrating the articular surface (6 versus 0, *p* = 0.027) significantly differed (Figure 2); probing method showed the absolute advantage. During the 12-month follow-up, no one had nonunion. In the traditional group, 1 case had humeral head necrosis, 1 case had screw loosening, and 2 cases had screw penetration (Figure 3). In the probing group, 2 cases had screw loosening; 1 case had subacromial impingement (Table 1). There were no statistically significant differences in complication rates between patients treated with either traditional measuring method or probing with depth gauge. In the 12th month function evaluation, the score of traditional group was 76.30 ± 8.69, including 2 excellent, 13 good, 4 moderate, and 1 poor; the score of probing group was 77.05 ± 7.73, including 3 excellent, 13 good, and 4 moderate. The score (*p* = 0.775) and fineness rate (80% versus 75%, *p* = 1.000) showed no statistical differences (Table 2).

## 4. Discussion

Most displaced proximal humeral fractures can be treated with locking plate. Locking plate has made a great progress in treating displaced proximal humeral fractures, which makes the joint-preserving surgery method more successful in elderly osteoporotic patients [22]. Compared to standard plates, locking plates were found to provide better stiffness and torsional fatigue resistance [23], as well as significantly greater holding power of the humeral head [24]. In theory locking plate has a lower rate of implant failure or fixation loosing due to more stable fracture fixation, thus allowing earlier mobilization. However, the overall complication rate remains high [9–12]. A retrospective analysis of locking plate in the treatment of proximal humeral fracture reported that the failure rate is as high as 13.7% [25]. In addition, intraoperative complications are also common; screw joint perforation is one of the most common complications, especially the iatrogenic penetration into the glenohumeral joint during operation [5, 13]. Kettler et al. [26] found that the incidence of intraoperative penetration of screws was 11%. Two other large series have reported intraoperative screw penetration rates of 11% and 14%, respectively [17, 18]. Although the penetrating screws are caused by secondary collapse or cutout [11, 27], we and some other authors believe that a certain proportion of penetration occurs during operation [10, 19, 28].

Several factors may result in the original penetration, such as the spherical surface of the humeral head and the diverging and converging characteristics of locking screws, which make assessing the position of the screw tip difficult on orthogonal views. Additionally the poor bone quality of osteoporosis patients limits the sensitivity of the tactile feedback of drill bit. So it is challenging to detect the screw penetration with the 2-dimensional C-arm fluoroscopy, even though the live fluoroscopy was taken during passive range of motion [28]. Strategies to minimize such complication are being developed in recent years. Weil et al. [29] introduced the use of C-InSight (Mazor Surgical Technologies, Caesarea, Israel), which allowed the use of a conventional 2-dimensional C-arm fluoroscope, coupled with a target array, to capture and produce 3-dimensional fluoroscopic images. More sophisticated intraoperative imaging devices have appeared that enabled producing 3-dimensional images, such as O-arm fluoroscopy [30]. These advanced devices were very useful in verifying the reduction of intra-articular fracture [30–32] but also assisted in detecting accidental destruction of articular surface by screws [33]. However, it was not routinely available in clinical practice, maybe because of the high cost and the large amount of radiation exposure during each scan [34].

However, the accidental penetration during operation, even when recognized and replaced, may disrupt the overall integrity of articular surface of the humeral head and more likely cause cutout and varus collapse. It is known that the subchondral bone of the humeral head offers the best grip, and it has a mean width of 5 mm [14]. For elderly patients, the quality of the subchondral bone is typically poor. As a result, it is difficult to distinguish the tactile feedback between cancellous bone and the thin subchondral bone during drilling. Therefore, the traditional measuring method with advancing the drill bit is unreliable, which may result in high accidental penetration rate.

To solve the above-mentioned problem, Bengard and Gardner [19] used a blunt-tipped Kirschner wire to verify the tactile feedback of the bone to get the screw tip location. In our study, we used a depth gauge instead of the Kirschner wire. When bone is osteoporoticly changed, cortical bone is thinner and cancellous bone is built of thin trabeculae, so the bone tissue is fragile due to lower density and changed trabecular built. As a result, the depth gauge can easily penetrate the cancellous bone by hand until the subchondral bone. Compared to the Kirschner wire, the depth gauge was easy to hold when it was advanced by hand and provided a more clearly tactile feedback; in addition, the depth can be measured directly. In our experience, the metal strength of the depth gauge was enough to penetrate the cancellous bone of osteoporotic patients to the subchondral bone without bending deformation, which was confirmed by the fluoroscopy. In addition, probing with depth gauge will decrease the disruption of the cancellous bone compared to the drill bit; thus the screw will have a better compression effect on the surrounding bone during screw insertion, like the PFNA blade, which improves the stability of the fixation. Therefore, even if the depth gauge tip cannot reach the subchondral bone in the patients with relatively hard bone, the compression effect may provide enough holding force to avoid looseness.

According to our own findings, even though there were no statistical differences between the two groups, operative time and intraoperative blood loss were less in the probing group, which showed the decreasing trend. The probing method just offered help to the procedure of getting the screw length, which may not significantly influence the overall operative time. Even so, the probing method showed obvious advantage in decreasing the X-ray exposure time. In traditional measuring method, more fluoroscopy times were needed to verify whether the screw-tip had penetrated the articular surface, even using the live fluoroscopy. Replacing improper locking screws would also increase exposure time. Through probing method, the ideal screw length can be quickly obtained without frequent fluoroscopy. At the same time, the probing method significantly decreased the risk of accidental perforation during drilling the screw path, which preserved the integrity of articular cartilage.

The other complications between two groups had no statistical differences. Compared to the traditional group, no screw penetration occurred in the probing group during the follow-up period. Even though there was no statistic difference, our results still showed some advantage in protecting the articular surface and the stability of screw fixation. The CMs is a common and objective evaluation index for the shoulder's function. Many factors may affect the functional outcome, such as fracture type, function before injury, and rehabilitation. According to our findings, the mean score and fineness rate in the probing group were both higher, indicating some advantage.

Although the follow-up time in our study was relatively short, the results demonstrate several benefits of the probing method with depth gauge. A limitation of the study is the small number of patients; future study with large sample may have a better result. Another limitation is that our probing method with depth gauge is more applicable to geriatric patients with osteoporosis. It will bring difficulty to advance the depth gauge to the ideal position in the younger patients with strong bone quality, so patient selection is important.

## 5. Conclusions

Given the characteristics of osteoporosis, probing with depth gauge is an effective method to get the accurate depth of locking screws to humeral head. Probing method with depth gauge significantly decreased the radiation exposure and effectively avoided the accidental penetration during operation. We therefore recommend the use of the probing method with depth gauge in the treatment with locking plate for proximal humeral fractures of osteoporotic patients.

## Figures and Tables

**Table 1:** Intraoperative characteristics.

Parameters	Traditional group (*n* = 20)	Probing group (*n* = 20)	*p* value
Operative time (min)			0.217
Range	96–135	90–120	
Mean ± SD	110.55 ± 9.36	107.05 ± 8.22	
Blood loss (mL)			0.322
Range	90–220	90–200	
Mean ± SD	156.50 ± 33.13	146.50 ± 29.78	
X-ray exposure time (s)			0.011^*∗*^
Range	9–16	8–15	
Mean ± SD	13.15 ± 2.13	11.50 ± 1.73	
Original penetration, *n* (%)	6 (30%)	0	0.027^*∗*^

^*∗*^Statistically significant difference (*p* < 0.05).

**Table 2:** Postoperative characteristics.

Parameters	Traditional group (*n* = 20)	Probing group (*n* = 20)	*p* value
Complications			
Humeral head necrosis, *n* (%)	1 (5%)	0	1.000
Screw loosening, *n* (%)	1 (5%)	2 (10%)	1.000
Screw penetration, *n* (%)	2 (10%)	0	0.468
Subacromial impingement, *n* (%)	0	1 (5%)	1.000
CMs			0.775
Range	53–90	59–92	
Mean ± SD	76.30 ± 8.69	77.05 ± 7.73	
CMs grades, *n *(%)			
Excellent (86–100)	2 (10%)	3 (15%)	
Good (71–85)	13 (65%)	13 (65%)	
Moderate (56–70)	4 (20%)	4 (20%)	
Poor(0–55)	1 (5%)	0	
CMs fineness rate (71–100), *n *(%)	15 (75%)	16 (80%)	1.000

CMs: Constant and Murley score.
